# Anatomic Changes in the Macroscopic Morphology and Microarchitecture of Denervated Long Bone Tissue after Spinal Cord Injury in Rats

**DOI:** 10.1155/2014/853159

**Published:** 2014-07-20

**Authors:** Ariane Zamarioli, Daniel A. Maranho, Mariana M. Butezloff, Patrícia A. Moura, José Batista Volpon, Antônio C. Shimano

**Affiliations:** ^1^Department of Biomechanics, Medicine and Rehabilitation, School of Medicine of Ribeirão Preto, University of São Paulo, Avenida Bandeirantes 3900, 14049-900 Ribeirão Preto, SP, Brazil; ^2^Laboratory of Bioengineering, School of Medicine of Ribeirão Preto, University of São Paulo, Pedreira de Freitas, Casa 1, Avenida Bandeirantes 3900, 14049-900 Ribeirão Preto, SP, Brazil

## Abstract

To study the effects of mechanical loading on bones after SCI, we assessed macro- and microscopic anatomy in rats submitted to passive standing (PS) and electrical stimulation (ES). The study design was based on two main groups of juvenile male Wistar rats with SCI: one was followed for 33 days with therapies starting at day 3 and the other was followed for 63 days with therapies starting at day 33. Both groups were composed of four subgroups (*n* = 10/group): (1) Sham, (2) SCI, (3) SCI + PS, and (4) SCI + ES. Rehabilitation protocol consisted of a 20-minute session, 3x/wk for 30 days. The animals were sequentially weighed and euthanized. The femur and tibia were assessed macroscopically and microscopically by scanning electronic microscopy (SEM). The SCI rats gained less weight than Sham-operated animals. Significant reduction of bone mass and periosteal radii was observed in the SCI rats, whereas PS and ES efficiently improved the macroscopic parameters. The SEM images showed less and thin trabecular bone in SCI rats. PS and ES efficiently ameliorated the bone microarchitecture deterioration by thickening and increasing the trabeculae. Based on the detrimental changes in bone tissue following SCI, the mechanical loading through weight bearing and muscle contraction may decrease the bone loss and restore the macro- and microanatomy.

## 1. Introduction

Spinal cord injury (SCI) initiates a cascade of systemic effects that disrupts the normal neural, vascular, hormonal, mechanical, and molecular balance, resulting in bone loss [1]. More than 50% of people with complete SCI will experience a postinjury bone fracture at some point [2]. The risk of fracture in a SCI individual is twofold the risk of fracture in an able-bodied person [3]. In addition to the markedly decreased quality of life, the mortality after a lower bone fracture is estimated to be 78% higher for SCI patients [4] and has often been associated with medical complications [2, 5].

The loss of bone tissue varies according to the anatomic region [3], and the most frequently fractured sites are the distal segment of the femur and the proximal segment of the tibia, where the bone tissue is predominantly trabecular [2]. The bone mineral density declines by approximately 50% at the distal epiphyses of the femur and by 60% at the proximal epiphyses of the tibia within 3 to 4 years after SCI. Afterward, the bone density reaches a steady state. The bone loss occurs at a minor intensity in the shaft and reaches 35% in the femur and 25% in the tibia within the first 5 to 7 years after injury [3]. Therefore, the osteoporosis after SCI is mainly caused by the loss of trabecular bone quality, which can be characterized by decreased mechanical properties, altered chemical composition, and disarrangement of the bone tissue microarchitecture [6]. Although assessing the bone microarchitecture provides substantial information about the changes that occur following paralysis, macroscopic anatomic changes may also play an important role in bone loss and fracture determinants [7, 8]. It is known that bones have specific angulations and curvatures, which enhance their capacity to resist bending and shear stresses [7]. Furthermore, the periosteal radius is a major determinant of bone resistance against fracture [9], which might account for the elevated risk of bone fracture within the SCI population. McCarthy has demonstrated the morphological changes in bone following SCI, using peripheral quantitative computerized tomography. The authors found a decrease in the cross-sectional area and in the volumetric bone mineral density, which may be consistent with bone mechanical loss [10].

According to our previous experimental study, a significant loss of bone density and strength was found in rats after SCI [11], which may be secondary to the macro- and microstructural changes caused by the absence of weight bearing and muscle contraction. Additionally, we believe that reloading (either by passive standing (PS) or by artificial muscle contraction) may change both the macroscopic and microscopic anatomic structures of bone tissue from paraplegic rats. Thus, the present study assessed the macro- and microscopic anatomy of bones after complete SCI injury for the purpose of comparing bones without loading and bones after reloading using PS treatment and electrical stimulation (ES).

## 2. Materials and Methods

### 2.1. Spinal Cord Injury and Postoperative Care

Juvenile Wistar rats (weight, 200–225 g; age 7 wks) were anesthetized by the intraperitoneal (IP) administration of ketamine (87 mg/kg, IP) and xylazine (13 mg/kg, IP). Complete SCI was achieved by surgical transection of the cord at the T10 level, as previously described [11–14]. The Sham-operated animals underwent laminectomy with cord exposure but without cord transection and were used as the control group (Sham, *n* = 10). The animals were kept under standard laboratory conditions (room temperature 22 ± 2°C, humidity 55 ± 5%, 12 h light-dark cycles), housed in individual cages, and fed unrestrictedly with standard laboratory animal chow, containing 1.15% calcium and 0.88% phosphorus, and they were offered water* ad libitum*. The daily postoperative care consisted of a skin examination, neurological functional assessment, and bladder expression twice daily. All rats were administered (intramuscular) buprenorphine (0.03 mg/kg) twice daily for 5 days for postoperative pain and 10 mL Lactated Ringer's solution subcutaneously once daily for 5 days.

The experimental protocol and all of the animal care described herein were previously approved by our Institutional Animal Care and Use Committee.

### 2.2. Animal Groups

**Figure 1:**
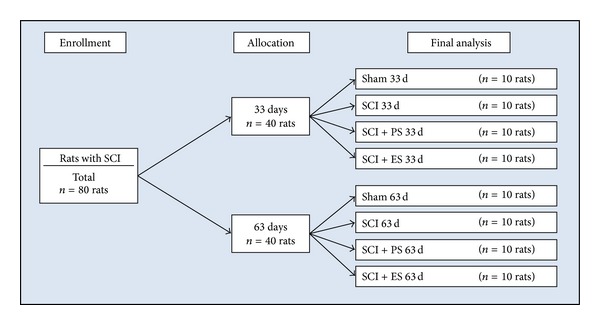
Flowchart of the experimental group design. (SCI: spinal cord injury; PS: passive standing; ES: electrical stimulation).

Eighty rats were divided into 8 groups (*n* = 10 per group, Figure 1). The first 4 groups were followed postoperatively for 33 days [(1) Sham 33 d, (2) SCI 33 d, (3) SCI + PS 33 d, and (4) SCI + ES 33 d], and the other 4 groups were followed postoperatively for 63 days [(5) Sham 63 d, (6) SCI 63 d, (7) SCI + PS 63 d, and (8) SCI + ES 63 d].

The control groups (33 d and 63 d Sham rats) consisted of animals that were able to ambulate normally; these animals were not submitted to physical therapy. The SCI group consisted of SCI animals that were not submitted to physical therapy. The SCI + PS group consisted of SCI animals that began PS treatment either on postoperative day 3 (SCI + PS 33 d) or on postoperative day 33 (SCI + PS 63 d). The SCI + ES group consisted of SCI animals that began electrical stimulation (to artificially contract denervated paralyzed muscles) either on postoperative day 3 (SCI + ES 33 d) or on postoperative day 33 (SCI + ES 63 d). Therapies that were initiated on postinjury day 3 were applied as a preventive physical modality against bone loss caused by the paraplegia. Therapies that were initiated on postinjury day 33 were applied as a therapeutic physical modality to revert the significant bone loss caused by the 30 days of paraplegia [11]. All treatments were performed 3 days per wk, 20 minutes/day for 30 days.

### 2.3. Passive Standing Treatment

The rats were placed in standing positions held by a custom-built static standing frame that maintained the hind limbs in extension, with the knees straight and the hind paws at a 90° angle with the level surface [11]. Positioning of the animal was optimized during each session to ensure an upright, erect position to avoid forward bending or rotation of the trunk.

### 2.4. Electrical Stimulation Therapy

Electrodes were placed over the motor points of the quadriceps and triceps surae muscles. Stimulation was performed with 300 *μ*s rectangular pulses delivered at 50 Hz with a 5 s on/15 s off duty cycle. The stimulation amplitude was adjusted (20 to 150 mA) to produce a concentric contraction that could be maintained for the stimulus duration (20 minutes).

The goal of the muscle contraction was to cause ankle plantar flexion and knee extension through the range of motion for each joint. Rats were not anesthetized during the therapy but were restrained inside a polyvinyl chloride tube.

### 2.5. Bone Assessment

At the end of each study time-point, the animals were weighed, which allowed the body mass to be compared at each time-point. The rats were then euthanized, and their bones were submitted to both macroscopic analysis and microscopic assessment by scanning electronic microscopy.

### 2.6. Macroscopic Analysis

The bones (tibias and femurs) were cleaned of soft tissues and then subjected to both qualitative and quantitative analyses. Qualitatively, the bones were compared among the groups, and then images were collected. Quantitatively, the bone mass, length, and perimeter were measured.

### 2.7. Scanning Electron Microscopy (SEM) Analysis

The distal femur and proximal tibia were sectioned in the coronal plane with a diamond-coated, low-speed saw, which measured 7 mm thick and 10 mm long. Sections were then washed repeatedly in distilled water, ultrasonicated, dehydrated in ascending grades of ethanol, and subjected to critical-point drying in CO_2_. The specimens were secured on aluminum stubs with conducting tape, coated with a thin layer of gold in a vacuum Bal-Tec SCD 050 (Fürstentum Liechtenstein) sputter coater, and studied with a Zeiss EVO MA10 (Cambridge, UK) scanning electron microscope in the secondary electron image (SEI) mode. Images were captured with an augment of 50x, whence the growth plate was used as the anatomical landmark for the superior limit of image.

### 2.8. Statistical Analysis

General linear models were used to compare the mean body weights and macroscopic bone measures at each site between each group. Tukey's correction was applied to adjust for multiple comparisons. The level of significance was set at 5%.

## 3. Results

### 3.1. Body Mass Reduction after SCI

**Figure 2:**
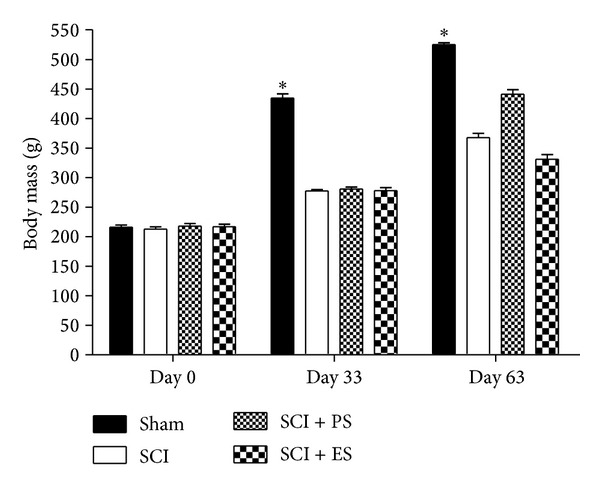
Body mass at each time-point of the study. At day 0, there was no difference among the groups; however, at both day 33 and day 63, the spinal cord injury (SCI) rats did not gain as much weight as the Sham animals. The asterisks indicate a statistically significant difference (*P* < 0.05). (PS: passive standing; ES: electrical stimulation). Error bars indicate standard deviation.

At study entry, the rat's body mass was similar among the groups (217 ± 15 g), indicating homogeneity among the animals (*P* > 0.05). At day 33, we observed that the SCI animals gained weight but not in the same proportion as the control group (30% versus 101%, *P* < 0.0001). The failure of the SCI rats to gain as much weight as the Sham rats persisted at the 63-daytime-point (73% versus 146%, *P* < 0.0001, Figure 2), whereas the body mass increased by 20% and 17% in the PS and ES groups, respectively, compared to age-matched SCI animals.

### 3.2. Macroscopic Anatomic Changes in the Femur

**Figure 3:**
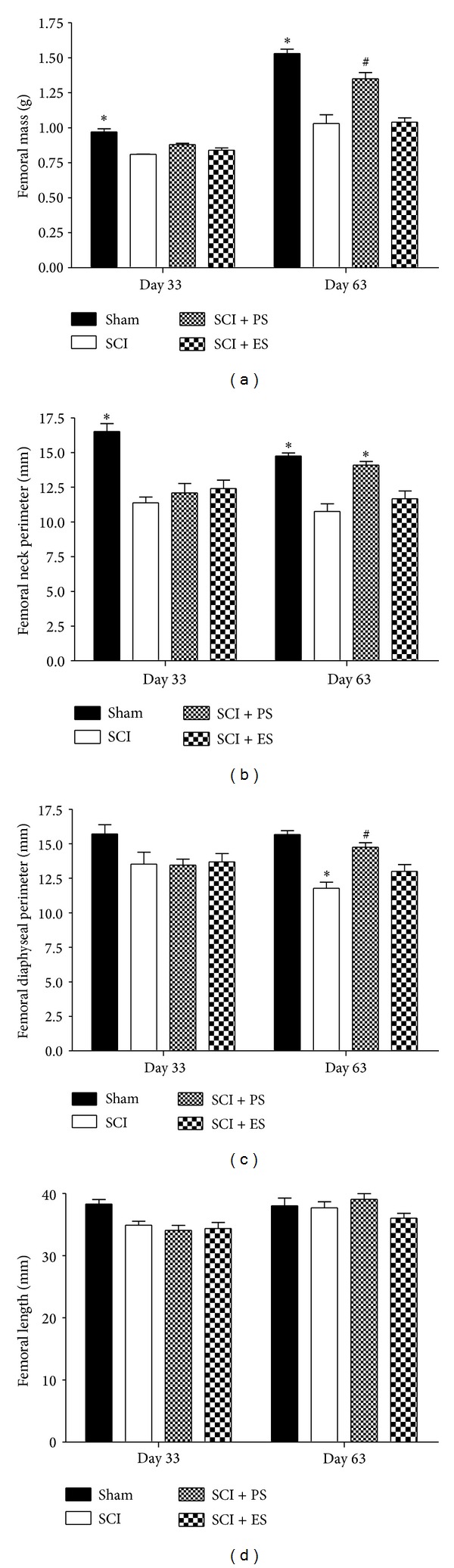
The macroscopic changes in the femur at 33 and 63 days after spinal cord injury (SCI). SCI rats showed deficits at increasing the bone morphometric measures to varying levels, whereas reloading through passive standing (PS) tended to reverse this loss and electrical stimulation (ES) slightly ameliorated it. The asterisks (∗) and hash (#) signs indicate a significant difference (*P* < 0.05). Error bars indicate standard deviation.

Bones from the paraplegic rats showed deficits at increasing morphological measurements at varying levels, whence these measurements were more pronounced after 63 days of injury (Figure 3). At 33 days, there was a significant reduction (16%) in the femoral mass, a 31% reduction in the femoral neck perimeter, and a 22% reduction in the diaphyseal perimeter (*P* < 0.05). At day 63, the reductions were 33% in the femoral mass, 27% in the femoral neck perimeter, and 25% in the diaphyseal perimeter (*P* < 0.05).

The PS treatment showed significant osteogenic effects not only at preventing bone loss but also at restoring the bone quality of the paraplegic rats. When applied in an acute stage (as a preventive therapy initiated 3 days after injury), PS significantly increased the femoral mass by 9% (*P* < 0.01). When applied in a chronic stage, to reverse the previously established bone loss, PS significantly increased the femoral mass by 36%, the femoral neck perimeter by 31%, and the femoral diaphysis perimeter by 25% (*P* < 0.01). The increase in the femoral neck perimeter was sufficient to completely restore its value to that considered to be normal (*P* > 0.05 in the Sham versus SCI + PS groups).

Although ES was not associated with significant prevention or reversion of the morphometric bone losses, nonsignificant (*P* > 0.05) increases of 31% in the femoral mass, 9% in the femoral neck perimeter, and 10% in the diaphysis perimeter were found when ES was applied in a chronic stage.

**Figure 4:**
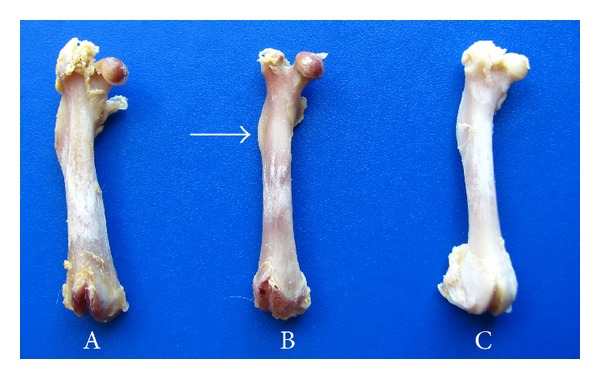
Anterior view of the right femur from 3 different groups: Sham (A); SCI (B); and SCI + PS (C). The lack of a mechanical load throughout both weight bearing and muscle contraction activities in the SCI rats (B) caused femoral morphological changes (i.e., a reduced bone perimeter and prominence of the gluteal tuberosity, arrow), whereas such changes were ameliorated by PS (C). (SCI: spinal cord injury; PS: passive standing).

Morphological changes in the macroscopic anatomy of the femur were observed following SCI.  Figure 4 shows the femurs from the Sham 63 d, SCI 63 d, and SCI + PS 63 d groups. Regarding the macroscopic proportions of the normal femur (A), it was possible to estimate a reduced bone size after SCI over the gluteal tuberosity and the greater and third trochanter, whereas such deficits were not found in the same proportions after reloading through PS (C).

### 3.3. Macroscopic Anatomic Changes in the Tibia

**Figure 5:**
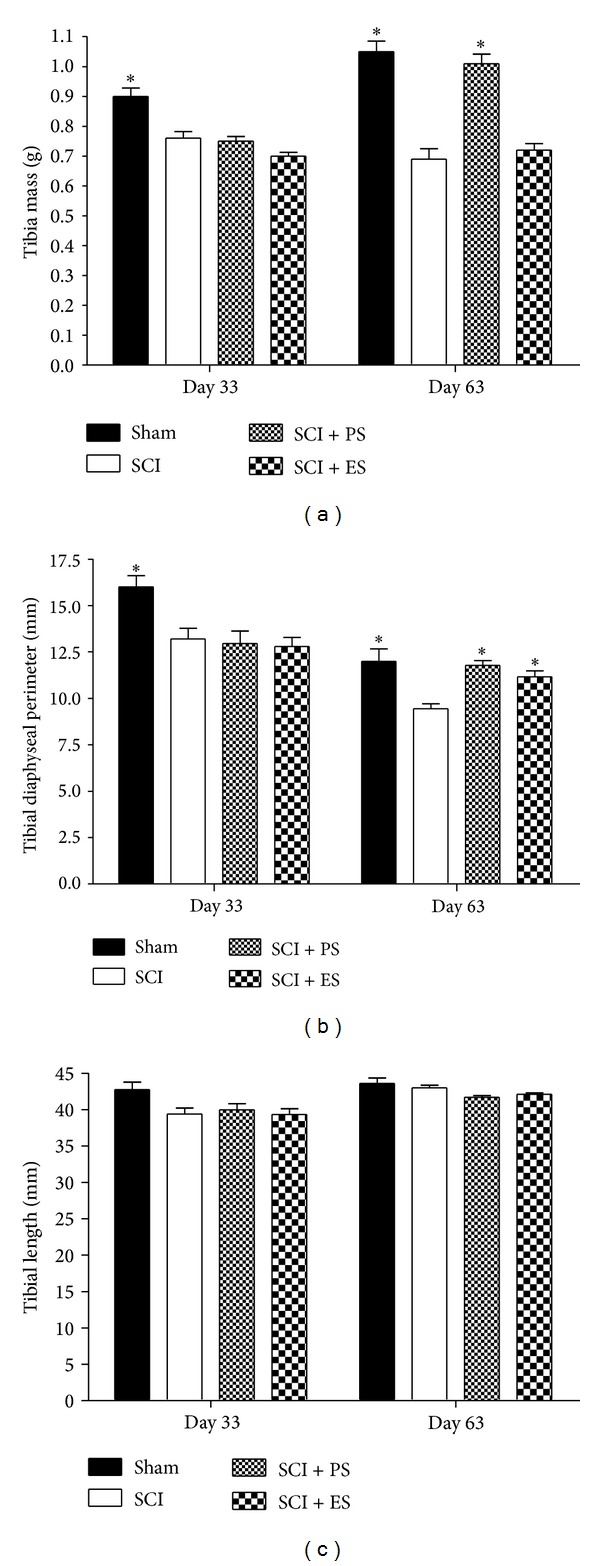
Macroscopic changes in the tibia at 33 and 63 days after spinal cord injury (SCI). SCI rats showed deficits at increasing the bone morphometric measures to varying levels, whereas reloading through passive standing (PS) reversed this loss and electrical stimulation (ES) also showed the same tendency. Asterisks (∗) and hash (#) signs indicate a significant difference (*P* < 0.05).

Similar to the results observed in the femur, the tibias from the paraplegic rats also failed at increasing the morphological measurements, when compared to the weight-matched controls (Figure 5); these measurements were more pronounced after 63 days of injury. At day 33, significant decreases of 16% and 17% were found in the SCI rats for the tibial mass and the diaphyseal perimeter, respectively (*P* < 0.05). At day 63, the reductions were 34% in the tibial mass and 21% in the diaphyseal perimeter (*P* < 0.05).

Neither PS nor ES increased the morphometric parameters when they were applied as a prevention modality.

PS treatment in the chronic stage completely reversed the morphometric losses caused by the paraplegia, and normal values were restored (*P* > 0.05 in the Sham versus SCI + PS groups). PS increased the tibial mass and the tibial diaphysis perimeter in 46% and 25%, respectively, of the SCI rats (*P* < 0.01).

Although ES did not significantly prevent or reverse the morphometric bone losses, nonsignificant (*P* > 0.05) increases of 4% in the tibial mass and 15% in the tibial diaphysis perimeter were observed when ES was applied at the chronic stage.

**Figure 6:**
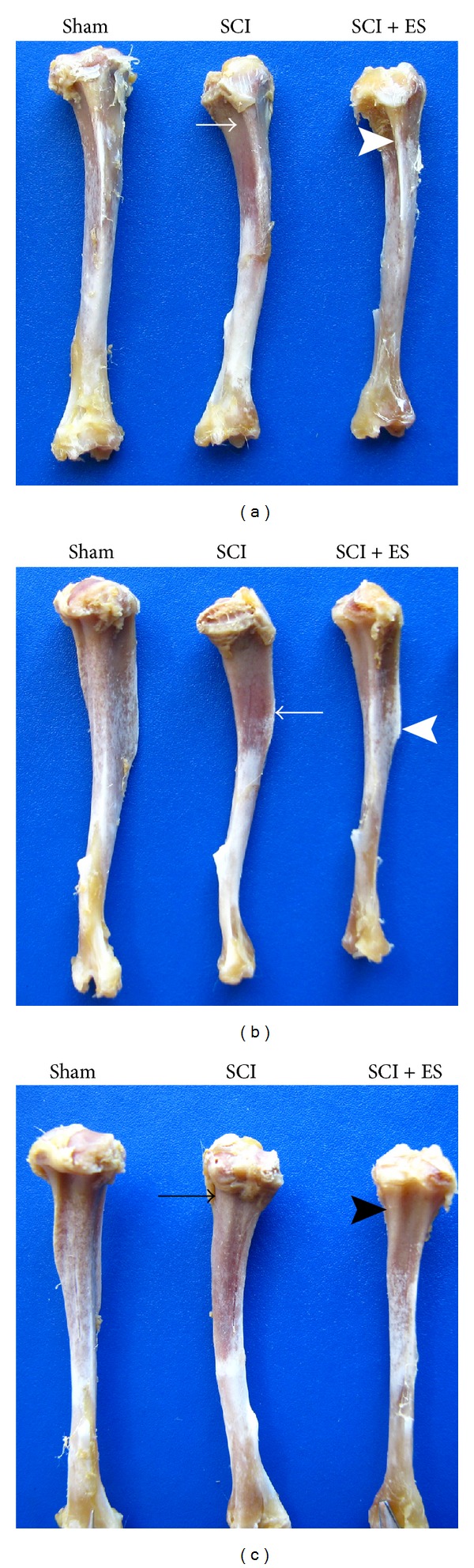
Anterior (a), lateral (b), and posterior (c) views of tibias from 3 different groups: Sham, SCI, and SCI + ES. The lack of muscle contraction caused anatomic changes in the tibias of the SCI rats. The anterior margin and tibial tuberosity are noticeably reduced in SCI (white arrows, (a) and (b)) when compared with Sham and SCI + ES. Conversely, ES stimulated bone formation and increased these anatomic prominences (white arrowheads, (a) and (b)). In the posterior view (c), the posterior tibial depression is practically nonexistent at the unloaded tibia (black arrow, SCI) but becomes more evident with muscle electrical stimulation (black arrowhead, SCI + ES). (SCI: spinal cord injury; ES: electrical stimulation).

Morphological changes in the macroscopic anatomy of the tibia were observed following SCI.  Figure 6 shows the tibias from the Sham 63 d, SCI 63 d, and SCI + ES 63 d groups. Regarding the macroscopic proportion of the normal tibia, it was possible to estimate a reduced bone size after SCI. In addition, the anterior tuberosity of the tibia was smaller in the SCI group, most likely because of the lack of patellar ligament traction. In group SCI + ES 63 d, the anterior tuberosity of the tibia was more evident, most likely because of the electrical stimulation of the quadriceps muscle.

### 3.4. Differences in the Bone Microarchitecture between the Unloading and Reloading Conditions

**Figure 7:**
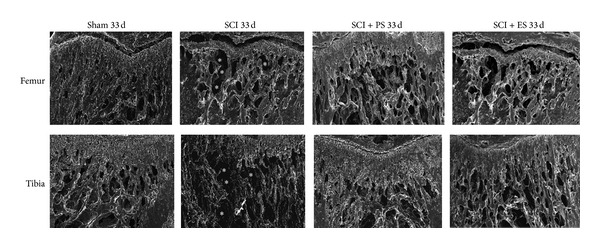
Microstructural changes in the femur and tibia at day 33 after unloading because of SCI and reloading by weight bearing (SCI + PS) and artificial muscle contraction (SCI + ES). Acute SCI increased bone resorption (asterisks) in both the tibia and the femur but was remarkably ameliorated by PS. (SCI: spinal cord injury; PS: passive standing; ES: electrical stimulation). Images were captured with an augment of 50x, whence the growth plate was used as the anatomical landmark for the superior limit of the image.

**Figure 8:**
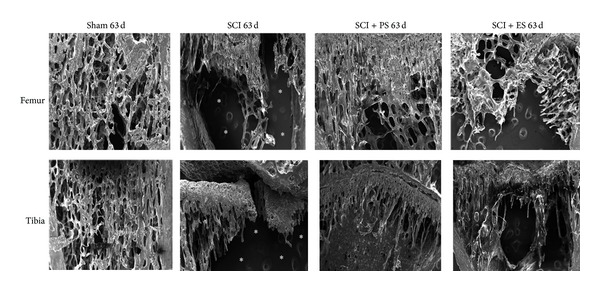
Microstructural changes in the femur and tibia at day 63 after unloading because of SCI and reloading by weight bearing (SCI + PS) and muscle electrical stimulation (SCI + ES). Chronic SCI substantially increased bone resorption (asterisks) in both the tibia and the femur but was mainly reversed by both PS and ES (to a lesser degree). (SCI: spinal cord injury; PS: passive standing; ES: electrical stimulation). Images were captured with an augment of 50x, whence the growth plate was used as the anatomical landmark for the superior limit of the image.

SEM images (Figures 7 and 8) showed intense changes in the bone microstructure in the femurs and tibias of the SCI animals, mainly on day 63. Mechanical loading by both weight bearing (SCI + PS) and muscle contraction (SCI + ES) improved the bone microstructure of the osteopenic bones, whereas the changes were greater following PS.

## 4. Discussion

Fractures in SCI patients have particular aspects that may differ from typical osteoporotic bone fractures because of the neurological deficit. Lower extremities bones in people with SCI can receive neither compression forces from weight bearing nor traction forces from muscle contraction. Thus, SCI induces substantial bone loss in the regions below the injury [2, 10, 11, 15]. In agreement with the literature, we found significant bone loss in paraplegic rats. This loss could be associated with the absence of mechanical loading and the lack of muscle contraction. At the macroscopic level, changes in the geometry and morphology were observed in the tibias and femurs of the paraplegic rats. Furthermore, the anatomic prominences of these bones became less evident after the SCI injury because of the absence of muscle contraction.

The lack of compression forces produces a greater negative impact at the trabecular bone of the epiphysis and metaphysis compared with the compact lamellar bone of the diaphysis, which is primarily subject to bending and torsional forces [16, 17]. Therefore, bone fractures in SCI individuals commonly occur at the metaphysis, which is a transitional zone between the condyles that has an extensive trabecular region with a thin cortical shell, than at the diaphysis, which has a thick cortical shell and almost no trabeculae [1, 15]. Previous studies have shown that the bone changes following SCI are more pronounced at the epiphyses [15, 17], which justifies our analysis of the bone microarchitecture at the femoral and tibial metaphysis.

It is known that the bone macroarchitecture influences bone resistance. The bone size and shape directly affect the bone strength, whereby the periosteal radius plays an important role in the bone resistance against bending loads [7, 9]. For instance, a modest increase of 8% in the periosteal radius causes a 36% increase in bone strength, which demonstrates that mechanical loading stimulates periosteal bone formation in areas under higher stress [9]. In our study, we observed macroscopic changes in the bone because of the absence of mechanical loading, which resulted in a reduced periosteal perimeter. The reduction at the periosteal perimeter was not only due to the absence of mechanical loading, but also because the rats were juvenile and still growing. Furthermore, because of the muscle paralysis, we found an overall reduction in the visible bone proportions and observed anatomic injuries (induced by SCI) within the macroscopic morphology of the bones. Morphological changes in the long bones caused by muscle contraction have also been demonstrated by previous researchers, who have used myostatin-deficient mice to show the direct relationship between increased muscle mass and bone formation [8, 18]. Conversely, no relationship has been found by other authors [19]. At the microscopic level, the bones of the SCI rats displayed marked trabeculae loss, leading to microarchitecture deterioration.

Many authors have studied the effects of PS and ES on the bone tissue in SCI patients. Some of these researchers have found that both PS [20–22] and ES [23–27] positively increased the bone quality after SCI. Conversely, previous studies have not demonstrated any significant effects of PS [28–31] or ES [32–34] at improving the bone quality following SCI. We previously found a positive effect of both PS and ES on the bone mineral density and bone strength in paraplegic rats [11]. As bone formation occurs preferentially in areas in which the strains are higher [9], we hypothesize that both PS and ES may stimulate local bone formation. In this study, we found that PS improved both the macro- and microarchitecture structure of the bone tissue after SCI. At the macroscopic level, the periosteal perimeter was substantially increased because of mechanical loading by weight bearing in the standing frame device. Thicker and more numerous trabeculae were found at the microscopic level, in addition to a higher trabecular organization. The artificial muscle contraction by ES also had positive macroscopic and microscopic effects on the bone tissue. At the macroscopic level, we found that quadriceps and triceps surae muscle stimulation partially restored the normal anatomic morphology at the tibias by stimulating bone formation at the muscle insertions [9]. At a microscopic level, we found an increase in the trabeculae number, thickness, and organization in the group subjected to the electrical stimulation treatment. Passive standing and electrical stimulation therapies ameliorated, and at some point reverted, the bone loss caused by the complete injury at the spinal cord. These findings confirm the important role of the mechanical loading on bone quality, by means of both weight bearing and muscle contraction. Although the improvement in bone quality was more pronounced in the animals submitted to passive standing, we may not infer that weigh bearing is better than muscle contraction at improving bone quality, since the exposure to weight bearing was longer than muscle contraction (in the ES therapy the “time off” is three times longer than the “time on,” which means that, during the 20 minutes of stimulation, muscle is really under contraction for less than 7 minutes).

## 5. Conclusions

We conclude that spinal cord injury causes detrimental changes in the macroscopic and microscopic anatomy of the bone tissue in paraplegic rats. Additionally, both mechanical loading through weight bearing and muscle contraction through electrical stimulation are efficient techniques at improving the normal anatomy of the long bones.
